# Hsp90aa1: a novel target gene of miR-1 in cardiac ischemia/reperfusion injury

**DOI:** 10.1038/srep24498

**Published:** 2016-04-14

**Authors:** Wen Si Zhu, Wei Guo, Jie Ning Zhu, Chun Mei Tang, Yong Heng Fu, Qiu Xiong Lin, Ning Tan, Zhi Xin Shan

**Affiliations:** 1 Guangdong Cardiovascular Institute, Guangdong General Hospital, Guangdong Academy of Medical Sciences, Guangzhou 510080, China; 2 Southern Medical University, Guangzhou 510515, China; 3 Guangdong Geriatrics Institute, Guangdong General Hospital, Guangdong Academy of Medical Sciences, Guangzhou 510080, China

## Abstract

The role of microRNA-1 (miR-1) in ischemia/reperfusion (I/R)-induced injury is not well illustrated. The present study aimed to investigate the expression and potential target of miR-1 in the myocardium of a rat model of I/R. The apoptosis of cardiomyocytes in the ischemic rat myocardium increased on day 1, then attenuated on day 3 and day 7 post-I/R. Heat shot protein 90 (Hsp90) aa1 mRNA expression was decreased post-I/R, and Hsp90aa1 protein level was decreased on day1 post-I/R, but was reversed on day 3 and day 7 post-I/R. MiR-1 was downregulated post-I/R, and repression of miR-1 in cultured neonatal rat ventricular cells (NRVCs) led to an increase of Bcl-2 and decreases of Bax and active caspase-3. Dual luciferase reporter assays revealed that miR-1 interacted with the 310–315 nt site at the 3′UTR of Hsp90aa1, and miR-1 was verified to inhibit Hsp90aa1 expression at the posttranscriptional level. Over-expression of Hsp90aa1 could attenuate oxygen-glucose deprivation (OGD)-induced apoptosis of NRVCs. Additionally, miR-1 mimic, in parallel to Hsp90aa1 siRNA, could enhance OGD-induced apoptosis of NRVCs. Taken together, our results reveal that Hsp90aa1 is a novel target of miR-1, and repression of miR-1 may contribute to the recovery of Hsp90aa1 during myocardial I/R.

Myocardial infarction remains an unsolved health problem, resulting in serious harm to human health. The ischemic myocardium is characterized by increased cardiomyocyte death, caused by necrosis and apoptosis. Reperfusion is essential for myocardial salvage after myocardial infarction; however, it is often followed by additional myocardial injury, termed myocardial ischemia/reperfusion (I/R) injury^1^,^2^,^3^. So far, the mechanisms of myocardial I/R injury has not been well illustrated. MicroRNAs (miRNAs) are endogenous, 20–23 nucleotide RNAs that negatively regulate gene expression at the post-transcriptional level^4^. Recent studies have indicated that miRNAs are widely involved in a variety of cardiovascular diseases, including arrhythmia, hypertrophy, heart failure and cardiac injury^5^. MicroRNA-1 (miR-1), is reportedly involved in the process of I/R injury. Some studies have demonstrated the up-regulation of miR-1 during I/R^6^,^7^,^8^, but others found miR-1 down-regulation^9^,^10^. Protein kinase C epsilon (PKCε)^7^, Bcl-2^8^, insulin growth factor 1 (IGF-1)^11^ and heat shock protein 60 (Hsp60)^7^,^12^ have been verified as target genes of miR-1 that mediate the effect of miR-1 in aggravating myocardial injury by I/R or infarction. Hsp90, one of the most abundant and conserved chaperones, helps preserve the integrity and function of numerous client proteins. Previous studies have shown that Hsp90 participates in ischemic preconditioning^13^,^14^,^15^, but its role in the process of myocardial I/R is not well known.

In this study, we hypothesized that Hsp90aa1 is a new target gene of miR-1 in cardiac ischemia/reperfusion injury. We investigated miR-1 expression in the rat myocardium post-I/R, and observed a significant decrease of miR-1 post-I/R. Furthermore, we demonstrated that rat miR-1 negatively regulated Hsp90 aa1 expression by directly targeting the 3′ untranslated region (UTR) of Hsp90 aa1 mRNA. Our data suggests that Hsp90aa1 is a novel target of miR-1 during myocardial I/R, and the recovery of Hsp90aa1 may be protective against myocardial I/R injury.

## Methods

### Ethics Statement

Male Sprague-Dawley (SD) rats weighing 250 ± 9 g, and 1- to 3-d old newborn SD rats, license number SCXK (YUE) 2004–0011 (Department of Experimental Animal Research Center, Sun Yat-sen University, Guangzhou, China) were used. The adult animals were housed under a 12-hr light/dark cycle under pathogen-free conditions, with free access to standard mouse chow and tap water. The animal experiments in the present study were carried out in accordance with the Guide for the Care and Use of Laboratory Animals as approved by the US National Institutes of Health (8th Edition, National Research Council, 2011).

### A rat model of I/R

I/R was induced by 45-min occlusion, followed by reperfusion of the left anterior descending coronary artery. Briefly, 35 rats were used to set I/R models and the Sham controls. Rats were anaesthetized with pentobarbital sodium at a dose of 35 mg/kg bodyweight intraperitoneally. The adequacy of rat anesthesia was obtained based on the absence of reflex response to foot squeeze. The rat body temperature was maintained during surgery at 37 ± 0.5 °C. Conditions of the animals were monitored every 15 min during surgery and every 2 to 3 hr after surgery. Thirty-two surviving rats that underwent ligation were randomly divided into three groups (N = 8): (1) I/R-1d, with 1-d reperfusion, (2) I/R-3d, with 3-d reperfusion and (3) I/R-7d, with 7-d reperfusion. The rats in the Sham group received an operation without LAD occlusion. At the end of the experiments, rats were sacrificed with an overdose of sodium pentobarbital (220 mg/kg, ip) and the rat myocardia was collected for further investigations.

### Terminal deoxynucleotidyl transferase dUTP nick end labeling (TUNEL) assay

Terminal deoxynucleotidyl transferase dUTP nick end labeling (TUNEL) assay was performed as in our previous report^12^. Briefly, the area in the rat left ventricular myocardium which underwent I/R was fixed overnight in 10% formalin. Samples were embedded in paraffin and cut into 4 μm thick sections, followed by mounting sections on normal glass slides. Neonatal rat ventricular cells (NRVCs) were cultured on coverslips and were fixed in 4% paraformaldehyde after experimental treatments. Heart tissue sections were permeabilized with 20 μg/ml Proteinase K, and NRVCs were permeabilized with 0.2% Triton X-100. Cy5-dUTP (Amersham, Piscataway, NJ, USA) was used to label DNA strand breaks in the apoptotic cells. The level of TUNEL-positive cells was detected by fluorescence microscopy, about 200 cells per field in five different visual fields were counted in this study. Heart tissue sections from five to eight rats in each group were used for TUNEL assay.

### Culture of primary cardiomyocytes and treatments

NRVCs were isolated from the hearts of 1- to 3-d old newborn SD rats as described previously^16^. Fifty nM miR-1 mimic, 50 nM Hsp90aa1 siRNA and 100 nM miR-1 inhibitor (Ribobio, Guangzhou, China) were transfected into NRVCs by oligofectamine reagent (Invitrogen, Carlsbad, CA). NRVCs were infected with the recombinant Hsp90aa1 adenovirus and the control GFP adenovirus vector at a multiplicity of infection (MOI) of 10, respectively. NRVCs were serum-starved overnight prior to all experiments. Cell ischemic injury was induced by oxygen-glucose deprivation (OGD) treatment, and was followed with reoxygenation as previously described^6^. Briefly, hypoxia was achieved by culturing NRVCs in serum- and glucose-free DMEM in a hypoxia chamber filled with 5% CO2 and 95% N2 at 37 °C for 4 hr. Then NRVCs were reoxygenated in DMEM containing 5% serum and normal glucose in a chamber filled with 5% CO2 and 95% O2 for 12 hr.

### Determination of mitochondrial membrane potential (ΔΨm)

At 24 h post-transfection of miR-1 mimic or miR-1 inhibitor, NRVCs were washed thrice with PBS (pH 7.2, 1 mL), then incubated for 15 min with 3 μM rhodamine 123 (Molecular Probes, USA) in PBS. Cell suspensions were incubated for 15 min at 37 °C. Cells were subsequently analyzed with a flow cytometer (Beckman, USA). Results were expressed as the proportion of cells exhibiting low mitochondrial membrane potential estimated by reduced uptake of rhodamine 123.

### Quantitative mRNA and miRNA measurements

Quantitative reverse transcription PCR (qRT-PCR) was performed as previously described^17^. Briefly, for detection of mRNA expression of coding genes, first-strand cDNAs were generated from 1.5 μg total RNA using a mixture of oligo (dT)_15_ and random primers with superscript reverse transcriptase (Invitrogen, Carlsbad, CA). qRT-PCR for miR-1 was performed on cDNA generated from 0.5 μg total RNA according to the manufacturer’s protocol (Ribobio, Guangzhou, China). To normalize RNA content, β-actin was used for coding genes template normalization and U6 was used for miR-1 template normalization. PCR and analyses were performed with a vii A7 Quantitative PCR System (Applied Biosystems, Carlsbad, CA). The 2-^∆∆Ct^ method was used to calculate relative expression levels of coding genes and miR-1 between treatments^18^. PCR primers for coding genes, miR-1 and U6 are shown in Supplementary Table 1.

### Western blot analysis

The amount of 40 μg protein prepared from rat myocardium or NRVCs was used in a standard Western blot analysis. The polyvinylidene fluoride (PVDF) membrane binding sample protein was incubated with a high affinity anti-Hsp90aa1 antibody (1:2000 dilution), anti-Hsp90b1 antibody (1:1000) (Abcam, Cambridge, MA), anti-Bax antibody (1:1000), anti-Bcl-2 antibody (1:1000) and anti-Caspase-3 antibody (1:1000) (Cell Signaling Technology, Danvers, MA), respectively. An anti-β-actin antibody (1:2000) (Santa Cruz Biotechnology, Santa Cruz, CA) was used to detect level of β-actin as an internal control. Proteins were visualized using the ECL Plus detection system (GE Healthcare, Waukesha, WI).

### Dual luciferase assay for Hsp90 target identification

As in our previous report^12^, the recombinant luciferase reporter plasmids containing the potential miR-1 binding site sequences of the Hsp 90aa1 and Hsp90b1 genes were prepared. Using a site-directed mutagenesis kit (TransGen, Beijing, China), the miR-1 binding site sequence CATTCC was replaced with CTAAGC to construct recombinant luciferase reporter plasmids containing the mutant potential miR-1 binding sequences.

Human embryonic kidney (HEK) 293 cells (3 × 10^5^ cells per well in 12-well plate) were co-transfected with 200 ng of recombinant luciferase reporter plasmid, 50 nM miR-1 mimic, and 20 ng of pRL-TK as an internal control (Promega, Madison, WI). Activities of firefly luciferase (FL) and Renilla luciferase (RL) were measured 24 hr after transfection, and the relative ratio of the FL /RL was used to indicate the miR-1-mediated knockdown of target genes.

### Statistical analysis

The data are presented as the means ± s.e.m. In each experiment, all determinations were performed at least in triplicate. Differences between experimental groups were analyzed using Student’s *t*-test. A value of *p* < 0.05 indicated significance.

## Results

### Expressions of miR-1, Hsp90aa1, Hsp90b1 and apoptosis-related genes in the myocardium post-I/R

**Figure 1:**
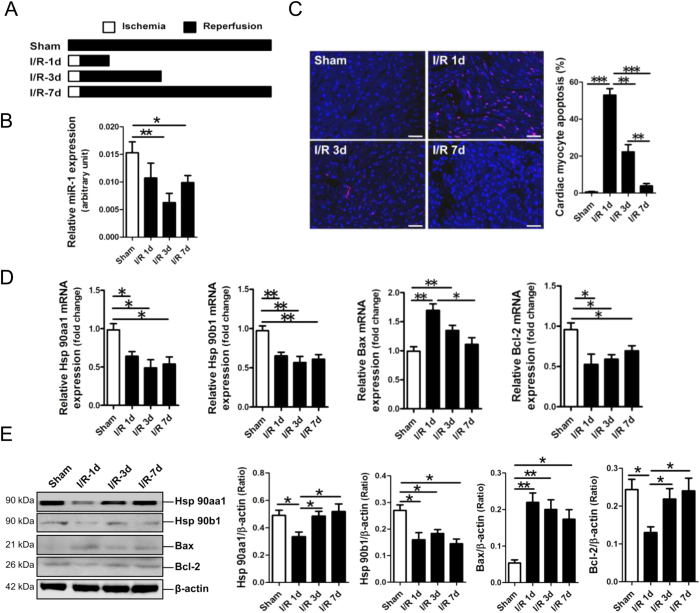
MicroRNA-1 (miR-1) expression in the myocardium of a rat model of ischemia/reperfusion (I/R). (**A**) Schematic outline of I/R surgery and experimental groups. (**B**) MiR-1 expression in rat myocardium by qRT-PCR assay. (**C**) The apoptosis of cardiomyocytes by terminal deoxynucleotidyl transferase dUTP nick end labeling (TUNEL) assay. The apoptotic cells are stained in red. The scale bar is 100 μm. (**D**) Hsp90, Bax and Bcl-2 mRNA expression in rat myocardium by quantitative reverse transcription-PCR (qRT-PCR) assay. (**E**) Hsp90, Bax and Bcl-2 protein expression in rat myocardium by Western blot assay. *p < 0.05, **p < 0.01, ***p < 0.001. N = 5–8.

TUNEL assay was performed in the four groups of rats — Sham, I/R-1d, I/R-3d and I/R-7d — to reveal cardiac apoptosis of rats in these groups (Fig. 1A). Results of qRT-PCR analysis showed that miR-1 was markedly decreased on day 3 and day 7 post-I/R (Fig. 1B). As expected, the apoptotic cardiomyocytes were dramatically increased in the myocardium of I/R-1d. Compared with I/R-1d, the apoptotic cardiomyocytes were markedly decreased in the myocardium of I/R-3d and I/R-7d (*p* < 0.01, *p* < 0.001) (Fig. 1C). Meanwhile, the apoptosis rate of cardiomyocytes in the myocardium of I/R-7d was lower than that in the myocardium of I/R-3d (*p* < 0.001) (Fig. 1C).

Moreover, we detected some altered gene expression in the myocardium of rat I/R models. MRNA expression of Hsp90aa1 was significantly decreased on day 1, day 3 and day 7 post-I/R (Fig. 1D). Hsp90aa1 protein was significantly decreased on day 1 post-I/R (*p* < 0.05), but was reversed on day 3 and day 7 post-I/R (*p* < 0.05) (Fig. 1E). Hsp90b1 mRNA and protein expression were significantly decreased on day 1, day 3 and day 7 post-I/R (*p* < 0.05, *p* < 0.01, respectively) (Fig. 1D,E). Bax mRNA was significantly increased in rat myocardium only on day 1 and day 3 post-I/R, but Bax protein expression was observed markedly increased on day 1, day 3 and day 7 post-I/R (*p* < 0.05, *p* < 0.01, respectively) (Fig. 1D,E). Bcl-2 mRNA expression was significantly reduced on day 1, day 3 and day 7 post-I/R (*p* < 0.05) (Fig. 1D), Bcl-2 protein expression was shown significantly decreased on day 1 post-I/R (*p* < 0.05), but was reversed on day 3 and day 7 post-I/R (*p* < 0.05) (Fig. 1E).

### Effect of miR-1 on apoptosis-related gene expression in rat cardiomyocytes

**Figure 2:**
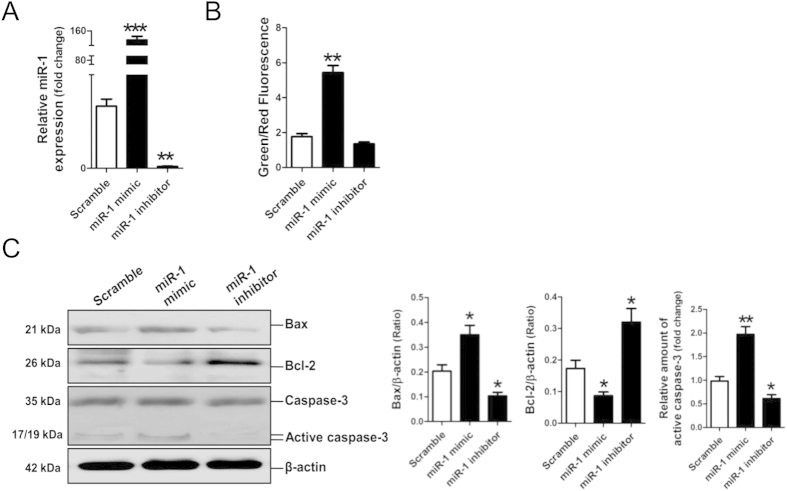
Apoptosis associated genes expression in cardiomyocytes after enforced expression of microRNA-1 (miR-1) mimic or miR-1 inhibitor. (**A**) MiR-1 level in NRVCs was detected by qRT-PCR assay. (**B**) Quantitative analysis of the shifts of mitochondrial potentials in NRVCs with transfection of miR-1 mimic or miR-1 inhibitor. An increase in the bar indicates a shift in the fluorescence ratio correlating with an increase in mitochondrial depolarization. (**C**) Bax, Bcl-2 and caspase-3 protein expression were detected by Western blot assay. *p < 0.05, **p < 0.01, ***p < 0.001. N = 3.

Fifty nM miR-1 mimic and 100 nM miR-1 inhibitor were transfected into NRVCs to assess the effect of miR-1 on apoptosis of cardiomyocytes. Results of qRT-PCR assay revealed that miR-1 was dramatically increased in miR-1-modified NRVCs, but was markedly decreased in miR-1 inhibitor-modified NRVCs (Fig. 2A). The mitochondrial potential assay indicated that the mitochondrial depolarization was significantly increased in NRVCs transfected with miR-1 mimic (Fig. 2B). In addition, results of Western blot assay showed that miR-1 significantly enhanced Bax expression and inhibited Bcl-2 expression; however, blockage of miR-1 function by miR-1 inhibitor significantly decreased Bax expression and increased Bcl-2 expression (all *p* < 0.05). Compared with the scramble control, miR-1 significantly increased the amount of the cleaved caspase-3, but miR-1 inhibitor significantly decreased the amount of the cleaved caspase-3 (*p* < 0.01, *p* < 0.05, respectively) (Fig. 2C).

### Verification of Hsp90aa1 as a target gene of miR-1

**Figure 3:**
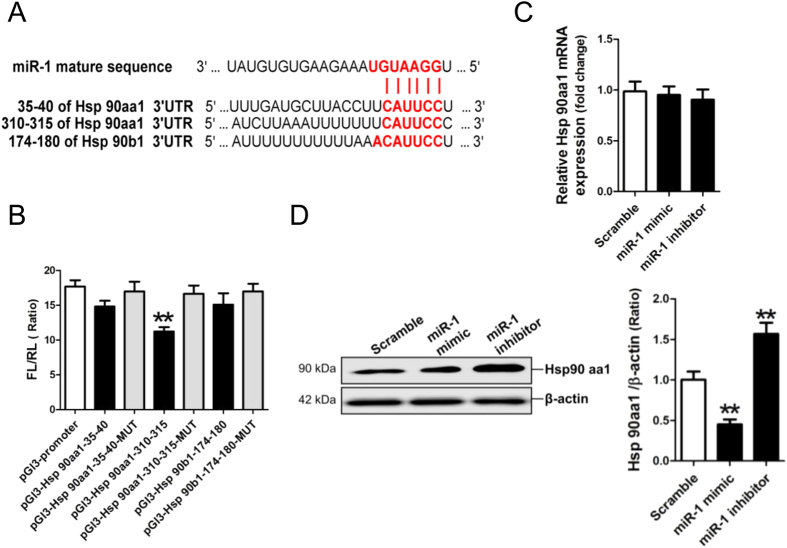
MicroRNA-1 (miR-1) negatively modulates Hsp90aa1 expression. (**A**) The predicted miR-1 seed sequence matches to potential target gene mRNAs. Results of *in silico* analysis suggest the presence of miR-1 target sites in the genes of Hsp90aa1 and Hsp90b1. The seed sequence of miR-1 is GGAAUGU, and the complementary nucleotide sequences are shown in bold. (**B**) Hsp90aa1 and Hsp90b1 verified as targets of miR-1 by the dual luciferase reporter system. Data on luciferase activity show the interaction between miR-1 and 3′UTRs of target genes. Data are shown as mean ± Standard Deviation, **p < 0.01 *vs* pGl3-promoter vector control, N = 3. MRNA expression (**C**) and protein expression (**D**) of Hsp90aa1 in miR-1 mimic and inhibitor-modified neonatal rat ventricular cells were assessed by quantitative reverse transcription-PCR assay and Western blot assay, respectively. Data are shown as mean ± Standard Deviation; **p < 0.01, N = 3.

Analysis of the databases Mirdb (www.mirdb.org) and TargetScan-Vert (www.targetscan.org) showed that Hsp90aa1 and Hsp90b1 were potential target genes of miR-1. The matching positions for miR-1 within 3′-UTR of the targeted mRNAs are shown in Fig. 3A. The dual luciferase assay demonstrated that miR-1 significantly reduced the luciferase activity through the 310–315 binding site, not the 35–40 binding site, in the 3′ UTR of the Hsp90aa1 gene, and not through the 174–180 binding site in the 3′ UTR of the Hsp90b1 gene (Fig. 3B).

Next, we detected the expression of Hsp90aa1 in NRVCs transfected with miR-1 mimic and miR-1 inhibitor, respectively. No significant change in Hsp90aa1 mRNA expression was found in miR-1 mimic- or miR-1 inhibitor-modified NRVCs (Fig. 3C). Hsp90aa1 protein expression was significantly reduced in miR-1 mimic-modified NRVCs, but was significantly increased in miR-1 inhibitor-modified NRVCs (*p* < 0.01) (Fig. 3D). Collectively, miR-1 inhibited the expression of Hsp90aa1, but not Hsp90b1, in NRVCs at the posttranscriptional level.

### MiR-1 and Hsp90aa1 siRNA enhanced the apoptosis of cardiomyocytes undergoing the oxygen-glucose deprivation (OGD) treatment

**Figure 4:**
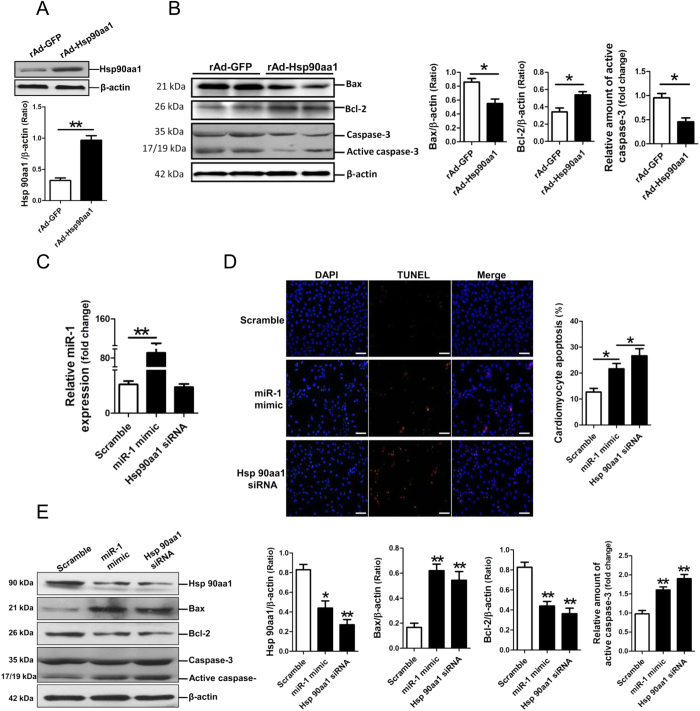
MicroRNA-1 (miR-1) inhibits Hsp90aa1 expression, contributing to apoptosis of cardiomyocytes under conditions of OGD. (**A**) Expression of Hsp90aa1 in NRVCs was detected by Western blot assay. (**B**) Expressions of Bax, Bcl-2 and active caspase-3 in OGD-treated NRVCs with over-expression of Hsp90aa1 by Western blot assay. (**C**) MiR-1 level in NRVCs with transfection of miR-1 mimic or Hsp90aa1 siRNA was detected by qRT-PCR assay. (**D**) Apoptosis of NRVCs by TUNEL assay. (**E**) Expressions of Hsp90aa1, Bax, Bcl-2 and active caspase-3 in OGD-treated NRVCs with transfection of miR-1 mimic and Hsp90aa1 siRNA, respectively. The scale bar is 100 μm. Data are shown as mean ± Standard Deviation, *p < 0.05, **p < 0.01. N = 3.

Hsp90aa1 expression was significantly increased in NRVCs through adenovirus delivery (Fig. 4A). NRVCs with enforced-expression of Hsp90aa1 were given OGD treatment. Compared with the NRVCs infected with adenovirus vector control, over-expression of Hsp90aa1 could attenuate OGD-induced apoptosis of NRVCs, with increasing the Bcl-2 level and decreasing the levels of Bax and active caspase-3 (Fig. 4B). MiR-1 mimic and Hsp90aa1 siRNA were transfected into NRVCs, followed by TUNEL assay and an examination of protein expression of apoptosis-related genes. MiR-1 level was markedly increased in NRVCs with transfection of miR-1 mimic (Fig. 4C), while Hsp90aa1 was significantly decreased in NRVCs with transfection of Hsp90aa1 siRNA (Fig. 4E). TUNEL assay results showed that miR-1 mimic and Hsp90aa1 siRNA consistently enhanced the OGD-promoted apoptosis of NRVCs (Fig. 4D). Western blot analysis demonstrated that Hsp90aa1 protein expression was consistently decreased in NRVCs with transfection of miR-1 mimic or Hsp90aa1 siRNA. Additionally, both miR-1 mimic and Hsp90aa1 siRNA significantly increased the levels of Bax and active caspase-3, and inhibited Bcl-2 expression in OGD-treated NRVCs (*p* < 0.01) (Fig. 4E). These results suggest that either miR-1 over-expression or the knockdown of Hsp90aa1 can similarly increase apoptosis of NRVCs exposed to OGD treatment.

## Discussion

Cardiomyocyte death is the direct and main cause of I/R-induced myocardial injury^19^. Bcl-2 and Bax are two important regulators of the mitochondrial apoptotic signaling pathway^20^, and Bcl-2 can prevent permeabilization of the outer mitochondrial membrane by inhibiting activation of Bax/Bak, leading to attenuation of the activation of caspase-9 and caspase-3^21^. HSP90 has been shown to participate in importing mitochondrial protein for hydrophobic membrane proteins, especially under stress conditions^22^. Previous studies have shown that Hsp90 plays a vital role in ischemic preconditioning^23^,^24^ and postconditioning^25^, and also mediates mitochondrial import of PKCε, helping to protect the myocardium against I/R injury^13^. As expected, our present study demonstrated that enforced-expression of Hsp90aa1 could attenuate, but knockdown of Hsp90aa1 could enhance OGD-induced apoptosis of cardiomyocytes (Fig. 2).

Hsp90 mRNA expression was reportedly up-regulated at 120 min and 180 min post-reperfusion^26^, however, Hsp90 expression for a longer period post-I/R is still not well known. In the present study, Hsp90aa1 mRNA and protein were observed decreased in rat myocardia on day 1 post-I/R, but Hsp90aa1 protein, not Hsp90aa1 mRNA, was reversed on day 3 and day 7 post-I/R. However, Hsp90b1 was shown decreased at both the mRNA and protein levels on day 1, day 3 and day 7 post-I/R. The above results indicate that the myocardial Hsp90aa1 is modulated at both the transcriptional level and the post-transcriptional level post-I/R.

MiRNAs have been demonstrated to be involved in myocardial I/R injury^27^. Here, we found that miR-1 was significantly down-regulated in rat myocardium on day 3 and day 7 post-I/R. Results of miR-1 expression patterns were inconsistent in myocardium undergoing preconditioning, post-conditioning and I/R treatment in previous reports^6^,^7^,^8^,^9^,^10^. Reasons for these uneven results include the differences in I/R conditions across studies, different time points of detection, cell types in the heart, as well as myocardium areas studied.

Previous studies revealed that miR-1 enhances apoptosis of cardiomyocytes by inhibiting the expression of anti-apoptosis genes, including PKCε^7^, Bcl2^8^, IGF-1^11^ and Hsp60^7^,^12^. As expected, in this study, we confirmed that over-expression of miR-1 enhanced the apoptosis of NRVCs. Bcl-2 was reported as a target gene of miR-1^8^. Consistently, the *in vitro* experimental data in this study revealed that inhibition of miR-1 resulted in increases of Bcl-2 (Fig. 2) and Hsp90aa1 (Fig. 3D), therefore, the significant decrease of miR-1 may contribute to recoveries of Hsp90aa1 and Bcl-2 protein in rat myocardia on day 3 and day 7 post-I/R.

Specifically, several lines of evidence derived from the current study support the notion that miR-1 negatively regulates Hsp90aa1 expression. The *in silico* prediction indicated that Hsp90aa1 and Hsp90b1 were potential targets of miR-1, however the results of dual luciferase assay showed that miR-1 specifically binds to the 310-315 site in the 3′-UTR of Hsp90aa1. In addition, miR-1 mimic could inhibit Hsp90aa1 protein expression, and miR-1 inhibitor could increase Hsp90aa1 protein expression, without significantly affecting Hsp90 aa1 mRNA expression. Moreover, in parallel with Hsp90aa1 siRNA, over-expression of miR-1 could also increase the levels of Bax and cleaved caspase-3, and suppress Bcl-2 expression in OGD-treated NRVCs, resulting in the enhancement of OGD-promoted apoptosis of cardiomyocytes.

Consistently, Hsp90aa1 and Bcl-2 were negatively modulated by miR-1 in rat myocardia post-I/R. Protein levels of Hsp90aa1 and Bcl-2 were decreased with no significant decrease of miR-1 on day 1 post-I/R, but were reversed with significant decrease of miR-1 on day 3 and day 7 post-I/R. However, mRNA levels of Hsp90aa1 and Bcl-2 were significantly decreased on day 1, 3 and day 7 post-I/R. The *in vitro* experimental data in this study revealed that inhibition of miR-1 resulted in increases of Hsp90aa1 (Fig. 3D) and Bcl-2 (Fig. 2), therefore, the significant decrease of miR-1 may contribute to recoveries of Hsp90aa1 and Bcl-2 protein in rat myocardia on day 3 and day 7 post-I/R.

Together, the present study demonstrated that miR-1 is decreased in rat myocardia undergoing I/R, and also identified that Hsp90aa1 is a novel target of miR-1. Attenuation of miR-1 may be required for recovery of Hsp90aa1 during myocardial I/R, moreover, suppression of miR-1 and recovery of Hsp90aa1 contribute to protection against myocardial I/R injury.

## Additional Information

**How to cite this article**: Zhu, W. S. *et al.* Hsp90aa1: a novel target gene of miR-1 in cardiac ischemia/reperfusion injury. *Sci. Rep.* **6**, 24498; doi: 10.1038/srep24498 (2016).

## Supplementary Material

Supplementary Information
