# Supplementary material for: Hsp90aa1: a novel target gene of miR-1 in cardiac ischemia/reperfusion injury
Source: Sci Rep. 2016 Apr 14;6:24498. doi: 10.1038/srep24498 (PMC4830926; doi:10.1038/srep24498)
Supplement: Supplementary Information [file srep24498-s1.pdf]

SREP-16-00775A

Title:

Hsp90aa1: a novel target gene of miR-1 in cardiac ischemia/reperfusion injury

Wen Si Zhu[1,2,*] , Wei Guo[3,*] , Jie Ning Zhu[1], Chun Mei Tang[2], Yong Heng Fu[1], Qiu Xiong Lin[1], Ning Tan [1] and Zhi Xin Shan[a,1]

[1] Guangdong Cardiovascular Institute, Guangdong General Hospital, Guangdong Academy of Medical Sciences, Guangzhou 510080, China

[2] Southern Medical University, Guangzhou 510515, China

[3] Guangdong Geriatrics Institute, Guangdong General Hospital, Guangdong Academy of Medical Sciences, Guangzhou 510080, China

[a] Email: zhixinshan@aliyun.com

[*] These authors contributed equally to this work.

Supplementary

Primers used in qRT-PCR assay

Gene	Sequence (5′- 3′)	Product size (bp）
Hsp90aa1	F, CCAACGACTGGGAAGAACAT	218
	R, CACCACCCCTCTGATGAAAT	
Hsp90b1	F, ATCTCCCCCTCAATGTTTCC	197
	R, AAGCCGTGTTCGATTTGAGT	
Bax	F, CCAGCTCTGAACAGATCATG	200
	R, CAATCATCCTCTGCAGCTCC	
Bcl-2	F, CGACTTTGCAGAGATGTCCA	198
	R, CACAGAGCGATGTTGTCCAC	
β-actin	F, GCCAACACAGTGCTGTCTG	203
	R, TACTCCTGCTTGCTGATCCA	
Mature miR-1	RT, GTCGTATCCAGTGCGTGTCGTGGAGT	76
	CGGCAATTGCACTGGATACGACATACACAC	
	F, GTCCGCTGGAATGTAAAGAAGTGTGTAT	
	R, GTGCGTGTCGTGGAGTC	
U6	RT, GTCGTATCCAGTGCGTGTCGTGGAGT	160
	CGGCAATTGCACTGGATACGAC	
	F, GTCCGCGTGCTCGCTTCGGCAGC	
	R, GTGCGTGTCGTGGAGTC	
